# The miRNA‐15b/USP7/KDM6B axis engages in the initiation of osteoporosis by modulating osteoblast differentiation and autophagy

**DOI:** 10.1111/jcmm.16139

**Published:** 2021-01-12

**Authors:** Xiaohui Lu, Yuantao Zhang, Yin Zheng, Bin Chen

**Affiliations:** ^1^ Department of Orthopedics The First Affiliated Hospital of Shantou University Medical College Shantou China; ^2^ Department of Teaching and Research The First Affiliated Hospital of Shantou University Medical College Shantou China

**Keywords:** autophagy, differentiation, KDM6B, microRNA‐15b, osteoporosis, USP7

## Abstract

Osteoporosis is a metabolic disease that results from oxidative stress or inflammation in renal disorders. microRNAs (miRNAs) are recently implicated to participate in osteoporosis, but the mechanism remains largely unexplored. Herein, we aimed to explore the potential role of miR‐15b in osteoblast differentiation and autophagy in osteoporosis. We established osteoporosis models through ovariectomy and determined that miR‐15b was highly expressed whereas USP7 and KDM6B were poorly expressed in tissue of osteoporosis mice. Treatment of silenced miR‐15b resulted in the elevation of decreased bone mineral density (BMD), the maximum elastic stress and the maximum load of osteoporosis mice. In osteoblasts, miR‐15 overexpression decreased proliferation but suppressed the cell differentiation and autophagy, accompanied with decreased expression of USP7. Mechanistically, miR‐15 bound and inhibited USP7 expression, while overexpression of USP7 promoted autophagy of osteoblasts. USP7, importantly, strengthened the stability of KDM6B and promoted KDM6B expression. MG132 protease inhibitor increased KDM6B and USP7 expression in osteoblasts. Silencing of KDM6B reversed the promoting effect on autophagy and proliferation induced by overexpression of USP7. Taken altogether, miR‐15b inhibits osteoblast differentiation and autophagy to aggravate osteoporosis by targeting USP7 to regulate KDM6B expression.

## 1 INTRODUCTION

Osteoporosis is regarded as a major public concern around the world, and more than 30% women over 50 years old are affected by the disease, around 50% of whom have a lifetime risk of fractures of the forearm, spine or hip, roughly the same as the risk of cardiovascular disease.^1^ Thus, prevention of osteoporosis has attracted great attention.^2^ It is associated with low bone mass, microarchitectural deterioration of bone tissue, increased bone fragility, leading to the increased risk of fragility fractures.^3^ In terms of pathogen, osteoporosis is mainly classified into primary, secondary and idiopathic osteoporosis. Primary osteoporosis characterized by bone fragility and decreased bone density is mainly affected by advanced age, sex‐steroid deficiency and increased oxidative stress.^4^ Secondary osteoporosis is usually induced by a drug, disease or deficiency while most known causes are Glucocorticoids, hypogonadism, alcohol abuse and malnutrition.^5^ Idiopathic osteoporosis tends to occur in young or middle‐aged males, usually associated with family history.^6^ It is reported that early diagnosis and treatment of osteoporosis are of great significance for the prevention of fracture in patients with osteoporosis.^7^ As we all know, in the absence of oestrogen, the excessive bone resorption of osteoclasts exceeds the bone formation of osteoblasts, resulting in the decrease of bone density, the destruction of bone microstructure, the imbalance of bone remodelling, and ultimately osteoporosis.^8^ Therefore, understanding the underlying mechanism involved in the osteoblast differentiation and autophagy processes is in urgent need for the treatment of osteoporosis.

Osteoporosis induced by ovariectomy is a classic primary type in which inhibition of autophagy decreased osteoclast differentiation with lower expression of osteoclast markers.^9^ Autophagy is a stress‐responsive catabolic process by which lysosomal enzymes degrade intracellular components, contributing to cellular and tissue homeostasis as well as metabolism.^10^ Autophagy promotes osteogenic differentiation and exerts its cytoprotective response in osteoblasts and bone marrow stem cells.^11^ To preserve the homeostasis, autophagy controls protein turnover and eliminates damaged macromolecules; however, autophagy disorder triggers the escape of quiescence, accompanied by alternations of transcription factors, enzymes, adhesion molecules and cytokines.^12^ Apart from autophagy, another prime degradation pathway in the cells is ubiquitin‐proteasome system, in which proteins tagged by polyubiquitin chains are removed by proteasome.^13^

microRNAs (miRNAs) are known as small noncoding RNA molecules that regulate gene expression, which are implicated in multiple biological processes.^14^ Abundant evidence has revealed that several miRNAs are reportedly involved in the regulation of the osteogenic differentiation and osteoblastic bone formation in osteoporosis.^15^ A recent study has elucidated that miRNAs exert effects on bone diseases such as osteoporosis.^16^ Moreover, miR‐15b is related to the occurrence of osteoporosis.^17^ The roles of miR‐15b in osteoblast differentiation in bone‐related defects and bone regeneration have also been demonstrated.^18^ Ubiquitin‐specific protease 7 (USP7), also regarded as Herpes virus‐associated USP or HAUSP, is a USP‐family DUB that regulates cellular targets and normal and disease biology.^19^ Tang et al have revealed that USP7 functions as a regulator in the progression of osteogenesis.^20^ However, the effects of miR‐15b regulating KDM6B by targeting USP7 on osteoporosis remain unclear. In the present study, the role of the miR‐15b in the osteoblast differentiation and autophagy in osteoporosis and its possible mechanisms are explored, which may help to provide a novel direction for treating osteoporosis.

## 2 MATERIALS AND METHODS

### 2.1 Ethics statement

This research study was conducted with the approval of the Ethics Committee of the First Affiliated Hospital of Shantou University Medical College. The animal experiment procedures were performed in accordance with strict principles of the Institutional Animal Care and Use Committee of the First Affiliated Hospital of Shantou University Medical College.

### 2.2 Bioinformatics analysis

miRNA expression profile GSE742009 and mRNA profile GSE56815 have been deposited in the publicly accessible database Gene Expression Omnibus (GEO; https://www.ncbi.nlm.nih.gov/gds). Profile GSE742009 contains 6 osteoporosis samples and 6 non‐osteoporosis samples, while profile GSE56815 contains 40 samples of high bone mineral density and 40 samples of low high bone mineral density. Differential analysis was performed using R language ‘limma’ (http://www.bioconductor.org/packages/release/bioc/html/limma.html) with a *P* value cut‐off of 0.05.

### 2.3 Establishment of osteoporosis mouse model

A total of 54 female C57BL/6J mice (aged 6‐8 weeks, weighing 22‐27 g) were provided by the Hunan SJA Laboratory Animal Co., Ltd (Changsha, China). The mice were raised in separate cages under 12 hours of light and 12 hours of dark cycle with the temperature of 22‐24℃, and free to food and water. Six mice were sham‐operated and injected with 0.2 mL of Phosphate‐Buffered Saline (PBS), and 48 mice were subjected to ovariectomy to establish oestrogen deficiency mouse osteoporosis models.^21^ With 6 ovariectomized mice as controls, the other ovariectomized mice were injected with agomir‐NC (7 mg/kg), miR‐15b agomir (7 mg/kg), antagomir‐NC, miR‐15b antagomir, agomir‐NC + oe‐NC, miR‐15b agomir + oe‐NC and miR‐15b agomir + oe‐KDM6B plasmids (n = 6, each group), via anionic liposome (Invivofectamine 2.0, Life Technologies) from day 1 to day 3 during the first, third and fifth weeks. The plasmid expression vectors pcDNA3.1 were provided by Biomics Biotechnologies Co., Ltd. (Nantong, China) (Table S1).

The mice were sacrificed, and the bilateral femurs were separated from the hip joint and the knee joint with surgical scissors to remove the muscle and fascia, followed by measurement of the bone mineral density (BMD), the maximum elastic stress and the maximum load. A part of the femur tissues was stored in refrigerator at −20℃ for molecular measurement. The rest of the femur tissues were fixed with 4% neutral formaldehyde overnight at 4℃, decalcified by EDTA for 2 weeks, embedded in paraffin, sectioned and stained with haematoxylin and eosin (HE) staining.

Measurement of the bone density: BMD of mouse left femur was detected using a dual‐energy X‐ray bone mineral density instrument (Faxitron ultrafocus 100, USA). Measurement of maximum elastic stress and the maximum load: mouse left femur was placed on the two columns of the material testing machine (Galdabini, SUN1000 V669, Italy) with space between less than 20 mm. The upper indenter of the machine approached and compressed the femur, with constant displacement speed of 0.1 mm/min until bone fracture. The experimental data were recorded every 10 seconds using SMD digital signal processing system, in which maximum elastic stress and maximum load were recorded.^22^

Bone histomorphometric parameters were determined according to the report of the American Society of Bone and Mineral Research Nomenclature Committee.^23^

### 2.4 In‐situ hybridization

The mouse femur tissues were embedded in paraffin and cut into 5‐mm‐thick sections. In situ hybridization was performed based on the previously published study.^24^ The hsa‐miR‐15b probe (Exiqon, Denmark) was incubated at a concentration of 0.2 mmol, and anti‐Digoxigenin‐AP Fab fragments were used at a dilution of 1:2000 (Anti‐digoxin, Roche). Sections were then stained with a nitro‐blue tetrazolium chloride/BCIP solution (5‐bromo‐4‐chloro‐30‐indolyphosphate p‐toluidine salt, Roche) and counterstained with nuclear fast red. The staining was analysed using a Nikon inverted microscope (ECLIPSE Ti‐U, Nikon, Japan). Three samples were stained from each group.

### 2.5 HE staining

The femur tissue samples of mice were washed with normal saline, then fixed in 4% paraformaldehyde for 30‐50 minutes, washed, dehydrated, cleared, soaked with wax, embedded and sliced. The tissue slices were dried in an incubator at 45℃, dewaxed, washed with gradient alcohol and then with distilled water for 5 minutes. Subsequently, the sections were stained with haematoxylin for 5 minutes, washed with running water for 3 seconds, differentiated with 1% hydrochloric acid ethanol for 3 seconds, and stained with 5% eosin for 3 minutes. The tissue sections were observed under the microscope.

### 2.6 Alizarin red staining

After cells were cultured for 21 days, cells were stained with Alizarin red using the Bone mineralization kit (sigma, St Louis, MO, USA) at pH 4.2 for 10 minutes, and then washed with distilled water. The mineralized nodule was examined under the phase contrast microscope.

Quantification of mineralization: Cells were then placed in PBS/Triton/HCl and sonicated. Cells were next incubated in 5 μL of HCl 6 mol/L overnight at 4℃. After centrifugation at 1500 × g for 5 minutes, the supernatant was transferred into a 96‐well plate, where the protein content was treated with calcium assay working solution. With ammonium hydroxide to each well, absorbance at 595 nmol/L was measured by a plate reader using the DC protein assay kit BioRad.^25^

### 2.7 Alkaline phosphatase (ALP) activity

The samples were cultured in fresh osteogenic differentiation medium containing 50 mol/L ascorbic acid‐2‐phosphate, 10 mmol/L β‐glycerine phosphate and 100 nmol/L dexamethasone for 14 days. ALP activity was detected according to the reagent kit (Beyotime Institute of Biotechnology, Shanghai, China) and observed under the microscope. Besides, cells were transferred to a 96‐well plate and the optical density (OD) at 405 nm was measured using a spectrometer. The ALP activity was then normalized to the DNA content using a Quant‐iT PicoGreen kit (Invitrogen, Car, Cal, USA).

### 2.8 Osteoblast isolation, treatment, and transfection

In aseptic condition, the soft tissues on the femur surface of osteoporosis mice were cleaned quickly. After removing attached connective tissue and periosteum, the soft tissues were chopped into fragments of 1 mm^3^ and placed in fresh osteogenic differentiation medium containing 50 mmol/L ascorbic acid‐2‐phosphate, 10 mmol/L β‐glycerol‐phosphate and 100 nmol/L dexamethasone. After trypsin digestion, the tissues were added with Dulbecco modified Eagle's medium (DMEM)‐F12 and blown into the cell suspension, inoculated into a T75 culture bottle (Thermo Fisher Scientific Inc, Waltham, MA, USA). After 2 days of culture, when the osteoblasts were covered more than 90% area of the bottom of the bottle, osteoblasts were digested with 2.5 g/L pancreatin, followed by ALP staining and alizarin red staining. Then the separated cells were identified as osteoblasts (Figure S1). The osteoblasts at logarithmic phase were digested with trypsin and seeded onto 6‐well plates with density of 1 × 10^5^ cells/well. After culture for 24 hours, when the cell confluence reached about 75%, cells were resuspended with serum‐free DMEM‐F12 medium, and then inoculated into 12‐well plates. Cells were transfected with mimic‐NC, miR‐15b mimic, inhibitor‐NC, miR‐15b inhibitor, oe‐USP7, and si‐KDM6B as well as administered with dimethyl sulphoxide (DMSO) which served as control solution or MG132, an effective and reversible protease inhibitor respectively according to the instructions of Lipofectamine 2000 (Invitrogen). The 500 ng of plasmid vector pcDNA3.1 with concentration (50 nmol/L) (Table S2) was purchased from Guangzhou RiboBio Co., Ltd. (Guangzhou, China), and MG132 was purchased from Shanghai Hongye Biotechnology Co., Ltd. (Shanghai, China).

### 2.9 EdU staining

The cells were seeded in each well of a 24‐well plate, and three replicates were set for each group. EdU was added into the culture medium to reach a concentration of 10 µmol/L and incubated in the incubator for 2 hours. After removal of the culture medium, cells were fixed in PBS solution containing 4% paraformaldehyde at room temperature for 15 minutes, followed by washing with PBS containing 3% BSA two times, incubation with PBS containing 5% Triton‐100 at room temperature for 20 minutes, and washing with PBS containing 3% BSA two times again. Subsequently, cells were incubated with Apollo^®^ 567 (100 µL) (Guangzhou RiboBio Co., Ltd., Guangzhou, China) at room temperature for 30 minutes without light exposure, washed with PBS containing 3% BSA for two times, stained with 4',6‐diamidino‐2‐phenylindole (DAPI) for 5 minutes, and rinsed with PBS three times. The stained cells were examined with a fluorescence microscope (Shanghai Pudan Optical Chemical Instrument Co., Ltd, Shanghai, China) to record the number of positive cells. For determination of the percentage of EdU‐positive cells, the number of red‐fluorescent cells (positive cells) was divided by the number of blue‐fluorescent cells (total cells). Three fields of vision were randomly selected for each well.

### 2.10 RNA isolation and quantification

The total RNA (2 μg) was extracted from the obtained tissues and cells by Trizol reagent (16096020, Thermo Fisher Scientific, Waltham, MA, USA). For miRNA detection, the total RNA was reverse transcribed into complementary DNA (cDNA) according to the instructions of miRNA First Strand cDNA Synthesis (Tailing Reaction) kit (B532453‐0020, Shanghai Sangon Biotech, Shanghai, China). For mRNA detection, the total RNA was reverse transcribed into cDNA according to the instructions of Reverse transcription Kit (RR047A, Takara Bio Inc, Otsu, Shiga, Japan). The cDNA was synthesized according to the instructions of cDNA Kit (K1622, Fermentas Inc, Ontario, CA, USA). Next, reverse transcription quantitative polymerase chain reaction (RT‐qPCR) was performed using TaqMan Gene Expression Assays protocol (Applied Biosystems, Foster City, CA, USA) with cDNA as template. β‐actin was used as an endogenous control. Three replicates were used for each treatment. Real‐Time PCR system (Applied Biosystems) was as follows: 95°C for 10 minutes, 40 cycles of 95°C for 10 seconds each and 60.5°C for 30 seconds. The primers used are listed in Table 1. Fold changes in gene expression were calculated by means of relative quantification (2^‐ΔΔCt^ method).

**TABLE 1:** Primer sequences for RT‐qPCR

Gene	Forward (5’→3’)	Reverse(5’→3’)
miR‐15b	CTCAACTGGTGTCGTGGAGTCGGCAATTCAGTTGAGTGTAAACC	ACACTCCAGCTGGGTTAGCAGCACATCAT
USP7	CGTTCGGAATCCCGTTTTTGCT	TCAAGGTAAGTGTAGCGACTCC
KDM6B	TGAAGAACGTCAAGTCCATTGTG	TCCCGCTGTACCTGACAGT
U6	CGCTTC GGCAGCACATATAC	TTCACGAATTTGCGTGTCAT
β‐actin	GTGGATCAGCAAGCAGGAGT	ATCCTGAGTCAAGCGCCAAA

### 2.11 Western blot analysis

The tissues and cells were lysed by PMSF lysis, split on ice for 30 minutes, and centrifuged at 10 000 rpm at 4℃ for 15 minutes. The supernatant was collected and transferred into the new Eppendorf tube. The protein concentration was measured with BCA Kit (Thermo Fisher Scientific). Next, the protein (30 μg) was separated by polyacrylamide gel electrophoresis and transferred onto a polyvinylidene fluoride (PVDF) membrane at a constant voltage of 80 V. After being blocked with 5% skim milk for 1 hour, the membrane was incubated with the primary rabbit antibodies at 4°C overnight, including USP7 antibody (ab4080, 1:1000), KDM6B antibody (ab38113, 1:b 1000), Runx2 antibody (ab23981, 1:1000), osterix antibody (ab22552, 1:1000), LC3Ⅱ/Ⅰ antibody (ab48364, 1:1000), P62 (ab10901, 1:10 000), and GAPDH antibody (ab181602, 1:10 000). All above antibodies were purchased from Abcam Inc (Cambridge, MA, USA). The membrane was then washed with PBST (PBS containing 0.1% Tween‐20) three times, 10 minutes per time. The membrane was then incubated with horseradish peroxidase (HRP)‐conjugated goat anti‐mouse IgG secondary antibody (ab6721, 1:2000, Abcam) at room temperature for 1 hour, followed by washing with PBST (PBS containing 0.1% Tween‐20) three times, 10 minutes per time. Finally, after scanning and developing with the optical luminescent instrument (GE, Niskayuna, USA), the relative expression of protein was analysed with the software of Image Pro Plus 6.0 (Media Cybernetics, NV, USA).

### 2.12 Dual‐luciferase reporter gene assay

The target gene analysis of miR‐15b was carried out by microrna.org to verify that USP7 was the direct target of miR‐15b. The synthetic USP7 3'UTR gene fragment was introduced into the pMIR‐reporter (Promega, Madison, WI, USA) using the endonuclease site SpeI and Hind II, and the complementary sequence mutation site of seed sequence was designed on USP7 wild type (WT). After restriction endonuclease digestion, T4 DNA ligase was used to insert the target fragment into the pMIR‐reporter plasmid. The dual‐luciferase reporter gene reporter plasmids of USP7 named as WT and mutant type (MUT) were constructed, both of which were then co‐transfected into HEK‐293T cells (Shanghai Beinuo Biology Co., Ltd. Shanghai, China) with miR‐15b, respectively. After 48 hours of transfection, the cells were collected and lysed, and luciferase activity was detected by luciferase test kit.

### 2.13 Co‐immunoprecipitation (Co‐IP)

The transfected cells were lysed in lysate buffer (mixture of 50 mmol/L Tris ‐ HCl (pH 7.4), 150 mmol/L NaCl, 10% glycerol, 1 mmol/L EDTA, 0.5% NP‐40 and protease inhibitor) and centrifuged to move cell debris. Cleared cell lysate was incubated with 1 μg anti‐HA (ab9110, 1:70, Abcam, Cambridge, UK), myc (ab32072, 1:100, Abcam, Cambridge, UK), USP7 (ab109109, 1:1000, Abcam, Cambridge, UK), KDM6B (ab38113, 1:100, Abcam, Cambridge, UK) or anti‐FLAG antibody (ab205606, 1:1000, Abcam, Cambridge, UK) and 15 μL protein A/G beads (Santa Cruz Biotechnology, Santa Cruz, CA, USA) for 2 hours. After extensive washing, the beads were boiled at 100℃ for 5 minutes. Proteins were resolved by sodium dodecyl sulphate‐polyacrylamide gel electrophoresis and transferred onto nitrocellulose membranes (Millipore, Temecula, CA, USA), followed by immunoblotting. To detect endogenous protein interactions, cells were lysed in ice‐cold lysis buffer. Cleared cell lysates were incubated with 5 μg anti‐USP7 antibody (ab4080, 1:1000, Abcam, Cambridge, UK) and 20 μL protein A/G beads at 4°C overnight. The anti‐USP7 antibody was used to detect the endogenous KDM6B.

### 2.14 Cycloheximide chase assay

USP7 and KDM6B were overexpressed in osteoblasts. At 36 hours after transfection, the cells were treated with 50 μg/mL cycloheximide. At different time points after treatment, the proteins extracted from the cells were collected and lysed. Western blot analysis was performed to determine KDM6B protein expression in different time points.

### 2.15 Statistical analysis

The SPSS 21.0 software (IBM Corp., Armonk, NY, USA) was used for all data analysis. All data were presented as mean ± standard deviation. The comparison between the two groups was performed using an unpaired *t* test. Data comparisons between multiple groups were performed by one‐way analysis of variance (ANOVA) with Tukey's post hoc test. The relationship between mir‐15b and USP7 was analysed using Pearson correlation analysis. A *P* value < .05 was considered to indicate a statistically significant difference.

## 3 RESULTS

### 3.1 miR‐15b was highly expressed in osteoporosis mice

**FIGURE 1:**
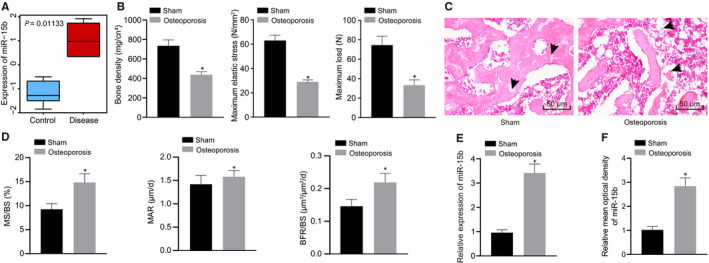
miR‐15b was up‐regulated in osteoporosis mice. (A) Box diagram of miR‐15b expression in microarray data GSE74209. The blue box on the left represents the samples of osteoporosis, and the red box on the right represents the samples of non‐osteoporosis. (B) BMD, maximum elastic stress and maximum load of osteoporosis mice. (C) Representative images of the histopathological changes of femur tissues of osteoporosis mice using HE staining (× 200). (D) Detection of dynamic osteogenesis parameters for femur by histomorphometry. (E) miR‐15b expression in femur tissues of osteoporosis mice determined using RT‐qPCR, normalized to U6. (F) In situ hybridization detection of miR‐15b expression. *P < .05 relative to sham‐operated mice. Data were presented as mean ± standard deviation. Data between two groups were analysed by unpaired *t* test. N = 6

We found that miR‐15b was significantly overexpressed in osteoporosis from microarray data GSE74209 in GEO database (Figure 1A). To further explore the expression and mechanism of miR‐15b in osteoporosis mice, osteoporosis mouse models were constructed by ovariectomy. Mice were then euthanized and their femurs were extracted. We observed that BMD, the maximum elastic stress and the maximum load of osteoporosis mice were lower than that of sham‐operated mice (Figure 1B). HE staining displayed the enlarged bone marrow cavity, thinned trabecular with fracture, and the widened gap between the trabeculae with irregular arrangement in the osteoporosis mice (Figure 1C). We then measured the dynamic bone formation parameters in histomorphometry and noticed that MS/BS and BFR/BS in the osteoporosis mice were significantly higher than that of sham‐operated mice (all *P* < .05) except MAR (*P* > .05) (Figure 1D). The above results revealed that the osteoporosis models were constructed successfully. RT‐qPCR and IHC experiment presented that miR‐15b was up‐regulated in the femur tissues of osteoporosis mice (Figure 1E, F).

### 3.2 miR‐15b overexpression inhibited osteoblast differentiation and autophagy to aggravate osteoporosis

**FIGURE 2:**
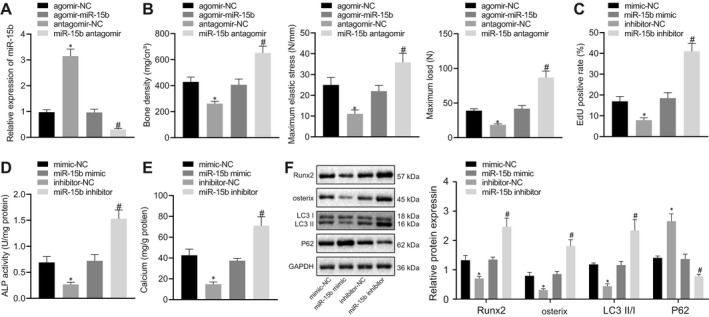
Up‐regulation of miR‐15b could suppress osteoblast differentiation and autophagy to aggravate osteoporosis. Osteoporosis mice were injected with miR‐15b agomir and miR‐15b antagomir (N = 6). (A) miR‐15b expression in femur tissues of osteoporosis mice determined using RT‐qPCR, normalized to U6. (B) BMD, maximum elastic stress and maximum load of osteoporosis mice. The osteoblasts were treated with exogenous miR‐15b mimic and miR‐15b inhibitor. (C) Proliferation of osteoblasts detected using EdU staining. (D) ALP activity of osteoblasts detected using ALP kit at day 21 after osteoblast differentiation. (E) The number of calcium nodules in osteoblasts using Alizarin red staining. (F) Protein levels of Runx2, osterix, LC3Ⅱ/Ⅰ, and P62 determined using Western blot analysis, normalized to GAPDH. *P < .05 relative to osteoporosis mice injected with miR‐15b antagomir or osteoblasts treated with mimic‐NC; # *P* < .05 relative to osteoblasts treated with inhibitor‐NC; data were presented as mean ± standard deviation. Data between multiple groups were analysed by one‐way ANOVA/Tukey's test

Osteoporosis mice were injected with miR‐15b agomir and miR‐15b antagomir to further explore the effect of miR‐15b on osteoporosis mice. RT‐qPCR displayed that miR‐15b expression increased in osteoporosis mice injected with miR‐15b agomir, but decreased in osteoporosis mice injected with miR‐15b antagomir (Figure 2A). Moreover, osteoporosis mice injected with miR‐15b agomir exhibited reduced BMD, maximum elastic stress and maximum load, while the results were opposite in osteoporosis mice injected with miR‐15b antagomir (Figure 2B). In addition, osteoblasts were treated with exogenous miR‐15b mimic and miR‐15b inhibitor. EdU staining revealed that cell proliferation was inhibited in osteoblasts treated with exogenous miR‐15b mimic, while proliferation was increased upon treatment with exogenous miR‐15b inhibitor (*P* < .05) (Figure 2C). ALP staining and Alizarin red staining displayed decreased ALP activity and number of calcium nodules in osteoblasts treated with exogenous miR‐15b mimic, while osteoblasts treated with exogenous miR‐15b inhibitor presented opposite trends (*P* < .05) (Figure 2D, E). Western blot analysis showed reduced protein levels of Runx2, osterix, LC3Ⅱ/Ⅰ and elevated P62 protein level in osteoblasts treated with exogenous miR‐15b mimic, but the results were opposite in osteoblasts treated with exogenous miR‐15b inhibitor (*P* < .05) (Figure 2F). These results suggested that restoration of miR‐15b inhibited osteoblast differentiation and autophagy to trigger osteoporosis.

### 3.3 Elevated miR‐15b inhibited osteoblast differentiation and autophagy to aggravate osteoporosis by negatively regulating USP7 expression

**FIGURE 3:**
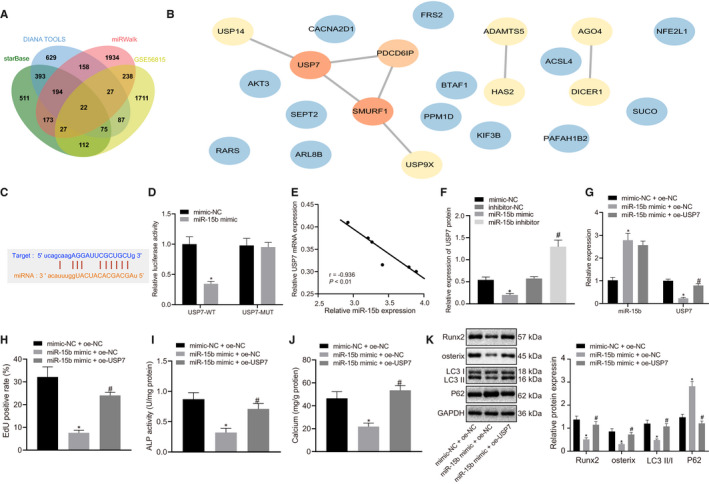
Up‐regulation of miR‐15b could suppress osteoblast differentiation and autophagy to aggravate osteoporosis by negatively regulating USP7 expression. (A) Venn map of 22 intersection genes between downstream genes of miR‐15b using starBase (clipExpNum > 5, pancancerNum > 1) (http://starbase.sysu.edu.cn/), DIANA TOOLS (miTG score > 0.7) (http://diana.imis.athena‐innovation.gr/DianaTools/), and miRWalk (accessibility < 0.01,au > 0.55) (http://mirwalk.umm.uni‐heidelberg.de/) and differentially expressed genes of microarray data GSE56815. (B) PPI network of intersection genes constructed String (https://string‐db.org/), the redder the circle of the gene, the higher the core degree; otherwise, the bluer the colour, the lower the core degree. (C) The target binding site between miR‐15b and 3'UTR of USP7 predicted using starBase. (D) Target relationship between miR‐15b and USP7 detected using dual‐luciferase reporter gene assay. (E) Correlation analysis of miR‐15b and USP7 expression. The osteoblasts were treated with exogenous miR‐15b mimic and miR‐15b inhibitor. (F) USP7 expression in osteoblasts determined using RT‐qPCR, normalized to β‐actin. (G) The transfection efficiency of miR‐15b and USP7 detected using RT‐qPCR. Osteoblasts were treated with exogenous miR‐15b mimic and oe‐USP7. (H) Proliferation of osteoblasts detected using EdU staining. (I) ALP activity of osteoblasts detected using ALP kit at day 21 after osteoblast differentiation. (J) The number of calcium nodules in osteoblasts using Alizarin red staining. (K) Protein levels of Runx2, osterix, LC3Ⅱ/Ⅰ and P62 determined using Western blot analysis, normalized to GAPDH. *P < .05 relative to osteoblasts treated with mimic‐NC or mimic‐NC + oe‐NC and # *P* < .05 relative to osteoblasts treated with inhibitor‐NC or miR‐15b mimic + oe‐NC. Unpaired *t* test was used to analyse data between two groups and one‐way ANOVA/Tukey's test to analysed data among multiple groups. The relationship between miR‐15b and USP7 was analysed using Pearson correlation analysis

In order to further study the downstream mechanism of miR‐15b, biological prediction website starBase, DIANA TOOLS and miRWalk predicted that there were 1507, 1585 and 2773 downstream genes of miR‐15b, respectively. On the other hand, 2299 genes with significant difference in osteoporosis were obtained by difference analysis of microarray data GSE56815 from GEO database. The intersection of downstream gene of miR‐15b and differentially expressed genes was identified to draw Venn map, which obtained 22 genes (Figure 3A). The PPI network of these 22 genes was constructed with String, and the core degree was calculated and plotted with Cytoscape (Figure 3B). We found that USP7 was the gene with the highest core and score (Table 2). According to the prediction of starBase, there was a target binding site between miR‐15b and 3'UTR of USP7 (Figure 3C). Dual‐luciferase reporter gene assay was used to further verify whether miR‐15b could target USP7, which showed that luciferase activity of WT‐USP7 3’UTR was significantly inhibited by miR‐15b (*P* < .05), but no difference was found in MUT‐ USP7 3’‐UTR (*P* > .05) (Figure 3D). Through the analysis of the correlation between miR‐15b and USP7, the results show that miR‐15b was negatively correlated with USP7 (*P* < .05) (Figure 3E). Then we transfected the osteoblasts with exogenous miR‐15b mimic and miR‐15b inhibitor, followed by Western blot analysis. It was clear that in the presence of miR‐15b mimic, USP7 expression in the cells was decreased whereas miR‐15b inhibition resulted in an increase in USP7 expression (Figure 3F). However, upon combined treatment of miR‐15b mimic and oe‐USP7, compared to miR‐15b mimic + oe‐NC treatment, osteoblasts exhibited lower level of miR‐15b expression and higher level of USP7 (Figure 3G). The above evidence indicated that miR‐15b inhibited USP7 expression in osteoporosis.

**TABLE 2:** The core degree and score of each gene in PPI network

Gene	Degree	Sum_combined_score	Gene	Degree	Sum_combined_score
USP7	3	1.747	CACNA2D1	0	0
SMURF1	3	1.308	SEPT2	0	0
PDCD6IP	2	0.863	ACSL4	0	0
DICER1	1	0.996	PAFAH1B2	0	0
AGO4	1	0.996	KIF3B	0	0
USP14	1	0.885	FRS2	0	0
USP9X	1	0.505	RARS	0	0
ADAMTS5	1	0.434	ARL8B	0	0
HAS2	1	0.434	PPM1D	0	0
NFE2L1	0	0	SUCO	0	0
AKT3	0	0	BTAF1	0	0

Note

Degree refers to the number of interaction between genes and other genes, and Sum_combined_score refers to the sum of scores of interaction between genes and other genes.

EdU staining, ALP staining and Alizarin red staining revealed promoted cell proliferation and increased ALP activity and number of calcium nodules in osteoblasts treated with exogenous miR‐15b mimic and oe‐USP7 (*P* < .05) (Figure 3H‐J). Western blot analysis showed elevated protein levels of Runx2, osterix, LC3Ⅱ/Ⅰ and reduced P62 protein level in osteoblasts treated with exogenous miR‐15b mimic and oe‐USP7 (*P* < .05) (Figure 3K). Taken altogether, miR‐15b might suppress osteoblast differentiation and autophagy, promoting osteoporosis progression.

### 3.4 USP7 enhanced the stability of KDM6B to promote KDM6B expression

**FIGURE 4:**
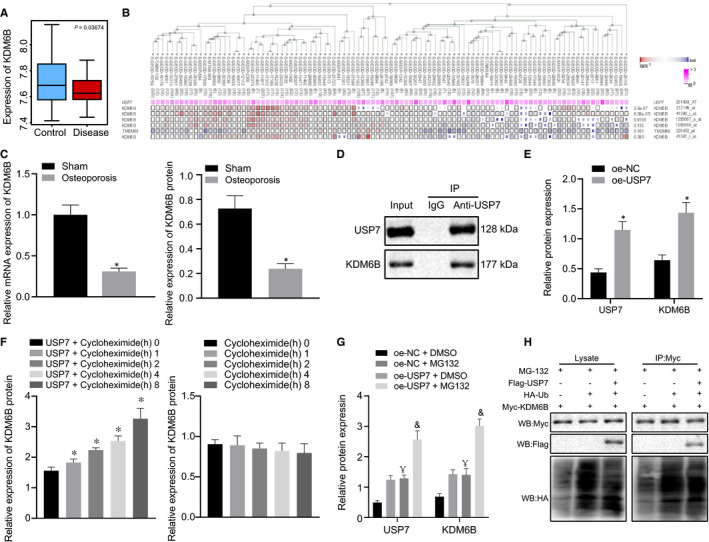
USP7 promoted the stability of KDM6B to promote KDM6B expression. (A) Box diagram of KDM6B expression in microarray data GSE56815. The blue box on the left represents the expression of high BMD samples, and the red box on the right represents the expression of low BMD samples. (B) MEM analysis co‐expression relationship between USP7 and KDM6B analysed using MEM (https://biit.cs.ut.ee/mem/index.cgi). (C) KDM6B expression in femur tissues of osteoporosis mice determined using RT‐qPCR and Western blot analysis, normalized to β‐actin. (D) Pull down of KDM6B protein by anti‐USP7 in the complex verified using IP experiment. (E) Protein levels of USP7 and KDM6B determined using Western blot analysis, normalized to GAPDH. (F) Stability of KDM6B regulated by USP7 detected using Cycloheximide chase assay. Osteoblasts treated with oe‐USP7 and protease inhibitor MG132. (G) KDM6B protein level determined using Western blot analysis, normalized to GAPDH. (H) Ubiquitination ability of KDM6B in osteoblasts. *P < .05 relative to sham‐operated mice or osteoblasts treated with oe‐NC or oe‐NC + DMSO, and & *P* < .05 relative to osteoblasts treated with oe‐USP7 + MG132. Unpaired *t* test was used to analyse data between two groups and one‐way ANOVA/Tukey's test to analysed data among multiple groups

It is found that KDM6B was significantly poorly expressed in osteoporosis through microarray data analysis in GEO database (Figure 4A). The co‐expression analysis of MEM verified that there was a significant relationship between USP7 and KDM6B expression (Figure 4B). RT‐qPCR and Western blot analysis revealed that KDM6B expression decreased in osteoporosis mice (*P* < .05) (Figure 4C). It was confirmed by IP experiment that anti‐USP7 could pull down the KDM6B protein in the complex, indicating the interaction between USP7 and KDM6B (Figure 4D).

We next further investigated whether the stability of KDM6B was regulated by USP7 using cycloheximide chase assay. Cells were overexpressed for KDM6B and USP7 expression and were treated with 50 μg/mL cycloheximide. Then cells were lysed to collect protein for Western blot analysis. It was observed that KDM6B protein level increased in osteoblasts treated with oe‐USP7 (*P* < .05) (Figure 4E) and overexpression of USP7 further elevated KDM6B protein level (Figure 4F). These data concluded that co‐expression of USP7 enhanced the stability of KDM6B to promote the KDM6B expression.

To further verify that USP7 affects KDM6B by ubiquitination, osteoblasts were transfected with oe‐USP7 or oe‐NC and then exposed to protease inhibitor MG132 and DMSO. Western blot analysis found that compared with oe‐NC and DMSO treatment, both MG132 + oe‐NC and oe‐USP7 + DMSO treatment increased USP7 and KDM6B protein expression, while combined treatment of oe‐USP7 and MG132 resulted in even higher KDM6B and USP7 protein level in osteoblasts (*P* < .05) (Figure 4G). Cycloheximide chase assay was performed applying anti‐USP7 antibody to pull down KDM6B. The results confirmed that the ubiquitination ability of KDM6B decreased in the presence of overexpressed USP7. It was speculated that the ubiquitination of KDM6B increased, that is, the expression of KDM6B increased significantly (*P* < .05) (Figure 4H). The obtained data implied that MG132 inhibited the ubiquitination of KDM6B by USP7 so as to enhance the stability of KDM6B and promote KDM6B expression.

### 3.5 USP7 promoted osteoblast differentiation and autophagy to ameliorate osteoporosis by increasing KDM6B expression

**FIGURE 5:**
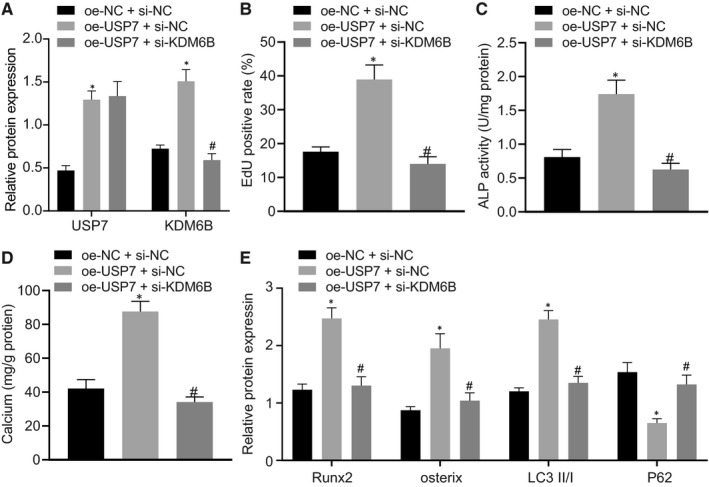
USP7 relieved osteoporosis differentiation and autophagy via up‐regulation of KDM6B. Osteoblasts were treated with oe‐USP7 and si‐KDM6B. (A) The transfection efficiency of miR‐15b and USP7 detected using Western blot analysis. (B) Proliferation of osteoblasts detected using EdU staining (× 200). (C) ALP activity of osteoblasts detected using ALP kit at day 21 after osteoblast differentiation. (D) The number of calcium nodules in osteoblasts using Alizarin red staining. (E) Protein levels of Runx2, osterix, LC3Ⅱ/Ⅰ, and P62 determined using Western blot analysis, normalized to GAPDH. *P < .05 relative to osteoblasts treated with oe‐NC + si‐NC) and # *P* < .05 relative to osteoblasts treated with oe‐USP7 + si‐NC. Data between two groups were analysed by one‐way ANOVA/Tukey's test

To identify the impact of interaction between USP7 and KDM6B on osteoblasts, we used vectors containing USP7, si‐KDM6B and control vector, whose inhibitory or mimicking activity was checked on osteoblasts (Figure 5A). EdU staining revealed that overexpression of USP7 enhanced cell proliferation of osteoblasts, which was restored by and the addition of si‐KDM6B (*P* < .05) (Figure 5B). ALP staining and Alizarin red staining displayed increased ALP activity and number of calcium nodules in osteoblasts treated with oe‐USP7, while osteoblasts treated with oe‐USP7 and si‐KDM6B presented opposite trends (*P* < .05) (Figure 5C, D). Western blot analysis showed elevated protein levels of Runx2, osterix, LC3Ⅱ/Ⅰ and reduced P62 protein level in osteoblasts treated with oe‐USP7, but the results were opposite in osteoblasts treated with oe‐USP7 and si‐KDM6B (*P* < .05) (Figure 5E). Therefore, the obtained data suggested that USP7 could induce osteoblast differentiation and autophagy to ameliorate osteoporosis by increasing KDM6B expression.

### 3.6 Up‐regulation of miR‐15b inhibited osteoblast differentiation and autophagy to aggravate osteoporosis by suppressing USP7/KDM6B axis

**FIGURE 6:**
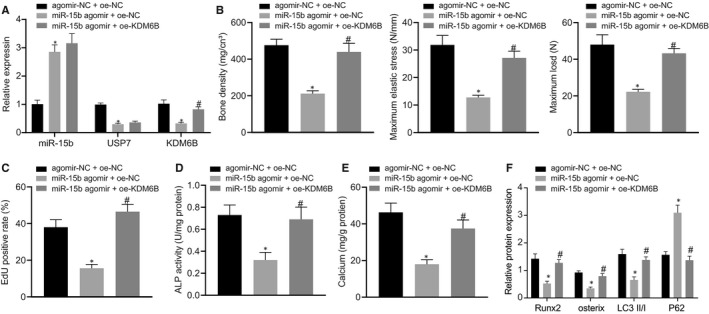
Restored miR‐15b inhibited osteoblast differentiation and autophagy to aggravate osteoporosis by suppressing USP7/KDM6B axis. Osteoporosis mice were treated with injected with miR‐15b agomir and expression vectors containing KDM6B (n = 6). (A) Expression of miR‐15b (normalized to U6), USP7 and KDM6B (normalized to β‐actin) in femur tissues of osteoporosis mice determined using RT‐qPCR. (B) Bone density, maximum elastic stress and maximum load of osteoporosis mice. The osteoblasts were treated with exogenous miR‐15b mimic and miR‐15b inhibitor. (C) Proliferation of osteoblasts in osteoporosis mice detected using EdU staining. (D) ALP activity of osteoblasts in osteoporosis mice detected using ALP kit at day 21 after osteoblast differentiation. (E) The number of calcium nodules of osteoblasts in osteoporosis mice using Alizarin red staining. (F) Protein levels of Runx2, osterix, LC3Ⅱ/Ⅰ, and P62 of osteoblasts in osteoporosis mice determined using Western blot analysis, normalized to GAPDH. *P < .05 relative to osteoporosis mice injected with agomir‐NC + oe‐NC and # *P* < .05 relative to osteoporosis mice injected with miR‐15b agomir + oe‐NC. Data between two groups were analysed by one‐way ANOVA/Tukey's test

To identify the in vivo interaction among miR‐15b, USP7 and KDM5B, we established models of osteoporosis mice and treated them with injection of miR‐15b agomir + oe‐NC, agomir‐NC + oe‐NC and miR‐15b agomir + oe‐KDM6B. Then the bones of mice were extracted and soft tissue on bone surface was collected for primary culture. RT‐qPCR displayed increased miR‐15b expression and decreased USP7 and KDM6B expression in osteoporosis mice injected with miR‐15b agomir, but reduced miR‐15b expression and elevated USP7 and KDM6B expression in osteoporosis mice injected with miR‐15b antagomir and expression vectors containing KDM6B (Figure 6A). Moreover, osteoporosis mice injected with miR‐15b agomir exhibited reduced BMD, maximum elastic stress and maximum load, while osteoporosis mice injected with miR‐15b antagomir and expression vectors containing KDM6B revealed increased BMD, maximum elastic stress and maximum load (Figure 6B). In addition, EdU staining, ALP staining and Alizarin red staining revealed inhibited cell proliferation and decreased ALP activity and number of calcium nodules of osteoblasts in osteoporosis mice injected with miR‐15b antagomir, but promoted cell proliferation and increased ALP activity and number of calcium nodules of osteoblasts in osteoporosis mice injected with miR‐15b antagomir and expression vectors containing KDM6B (*P* < .05) (Figure 6C‐E). Western blot analysis showed reduced protein levels of Runx2, osterix, LC3Ⅱ/Ⅰ and elevated P62 protein level in osteoporosis mice injected with miR‐15b antagomir, while the results were opposite in osteoporosis mice injected with miR‐15b antagomir and expression vectors containing KDM6B (*P* < .05) (Figure 6F). Thus, it can be concluded that miR‐15b overexpression suppressed osteoblast differentiation and autophagy to aggravate osteoporosis by suppressing USP7/KDM6B axis.

## 4 DISCUSSION

Initially, our results implied that miR‐15b was highly expressed in osteoporosis mice, which could inhibit osteoblast proliferation, differentiation and autophagy to aggravate osteoporosis. Moreover, bioinformatics analysis and dual‐luciferase reporter gene assay in combination validated that miR‐15b could target USP7 and suppresses USP7 expression. Additionally, the present study confirmed that USP7 enhanced the stability of KDM6B to promote KDM6B expression. Combining previous studies with our results, a regulatory network could be proposed in the antagonizing atherosclerosis progression that depletion of miR‐15b promoted USP7 expression to up‐regulate KDM6B.

miRNAs are important regulators of gene expression, with documented roles in osteoporosis, suggesting potential therapeutic targets.^16^ Deregulation of miRNAs is associated with multiple biological processes, such as proliferation, differentiation and apoptosis.^26^ Existing literature has reported that miRNAs have been described as critical factors regulating osteoblast biology including proliferation, differentiation and apoptosis in osteogenesis.^27^ miR‐15b is proved to be up‐regulated in osteogenesis,^17^ which is consistent with our findings. The effects of miR‐15b in osteoblast differentiation have also been investigated.^28^ It is interesting to note that miR‐34s serve as an inhibitor in osteoblast proliferation by decreasing levels of Cyclin D1, CDk4, and CDK6 and a suppressor in osteoblast differentiation by decreasing SATB2 to facilitate the progression of osteoporosis.^29^ Li et al have exhibited that miR‐133a expression elevated in was up‐regulated osteogenesis, which promoted osteoclast differentiation, indicating that knockdown of miR‐133a could alleviate the osteogenesis.^30^ However, there are few researches in the roles of miR‐15b in osteoporosis by regulating osteoblast proliferation, differentiation and autophagy. According to the abovementioned findings, it is a rationale to demonstrate that, silencing of miR‐15b could be considered as a potential therapeutic target to reverse osteoporosis. A recent paper showed that endothelial cell‐exosomes inhibit osteoclast activity in vitro and inhibit osteoporosis in an ovariectomized mouse model, so exosomal miR‐155 may have therapeutic potential against osteoporosis.^31^ It is likely that miR‐15b also exert similar effect, which remains elusive but deserves further investigation.

It has been implicated that USP7 acts as a modulator in cellular targets and normal and disease biology.^19^ A recent study has demonstrated that USP4 could promote osteoblast differentiation via Activation of Wnt/β‐catenin signalling by inducing Runx2.^32^ Accumulating evidence has also elucidated that up‐regulation of USP7 could enhance the osteogenic differentiation of human adipose‐derived stem cells (hASCs) to inhibit the progression of osteogenesis,^20^ suggesting that overexpression of USP7 could relieve atherosclerosis. Thus, these findings support that restoration of USP7 underlies the anti‐osteogenic effect of miR‐15b by stimulating osteoblast proliferation, differentiation, and autophagy.

Autophagy is key to bone homeostasis while autophagy dysfunction in bone cells is associated with the onset of bone diseases such as osteoporosis.^33^ The activation of autophagy significantly increased osteoblast differentiation activity and restored the bone volume.^34^ In ovariectomized mice, autophagy activation in osteoclasts is responsible for rapid bone loss and polarized secretion of lysosome.^11^ It has been noted that blocking of the ubiquitin‐proteasome system alleviates bone loss and osteoclast formation.^35^ Ubiquitination participates in positive feedback regulations for timely induction of autophagy, where the initiation and nucleation steps of autophagy are most prevalently regulated by ubiquitination.^36^ Of note, our data revealed that inhibition of proteasome up‐regulated expression of USP7 and KDM6B, while increased USP7 expression promoted osteoblast differentiation and autophagy, alleviating osteoporosis. The histone demethylases KDM4B and KDM6B play critical roles in osteogenic commitment of MSCs by removing H3K9me3 and H3K27me3.^37^, ^38^ In addition, a recent study has confirmed that USP7 could promote KDM6B expression by enhancing KDM6B stability.^39^ KDM6B, also named Jmjd3, emerged as a H3K27 demethylase that can catalyse the demethylation of H3K27me2/3 and is implicated in cellular processes, including differentiation, apoptosis and senescence.^40^ Knockdown of KDM6B significantly suppresses osteogenic differentiation to aggravate the osteoporosis, suggesting that KDM6B may present as therapeutic targets for promoting osteoblast differentiation and lead to clues for new treatment in osteoporosis.^37^ In this study, we found that USP7 enhanced the stability of KDM6B to promote KDM6B expression inhibited the ubiquitination, while miR‐15b inhibits USP7 expression in osteoporosis. Protein stability is the crux of advanced delivery technologies and media for targeted therapeutics.^41^ Its stability design and investigation are the opening to application of protein design methodology to large proteins and molecular activities.^42^ In osteoporosis, increased stability of protein inhibits ubiquitin‐proteasome‐mediated degradation, promoting alkaline phosphatase activity and bone mineralization.^43^

**FIGURE 7:**
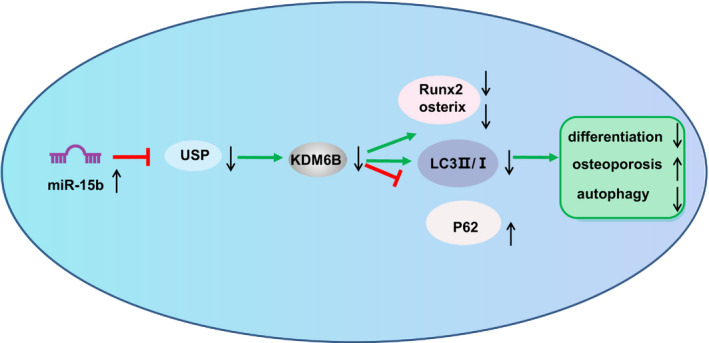
Schematic map of the mechanism of miR‐15b in osteoporosis. miR‐15b inhibits osteoblast differentiation and autophagy to aggravate osteoporosis by targeting USP7 to regulate KDM6B expression

In conclusion, our study demonstrated that up‐regulation of miR‐15b could inhibit the expression of USP7, which potentially suppress the osteoblast proliferation, differentiation and autophagy to aggravate osteoporosis through inhibition of KDM6B expression (Figure 7). Our study also provided further insight into the regulatory network and the underlying roles of miR‐15b regulating KDM6B via USP7 in atherosclerosis. However, this study did not investigate its possible influences of miR‐15b on other organs and cells, which is our limitation and also the direction of further experiment. Further investigations are required to evaluate its application in clinical trials in patients with osteoporosis. In addition, more studies are required in order to adequately define the detailed mechanisms by which miR‐15b interacts with USP7 and KDM6B and influences osteoporosis progression.

## CONFLICTS OF INTEREST

The authors declare that they have no conflict of interest.

## AUTHOR CONTRIBUTIONS

**Xiaohui Lu:** Conceptualization (equal); Writing‐original draft (equal); Writing‐review & editing (equal). **Yuantao Zhang:** Conceptualization (equal); Data curation (equal); Formal analysis (equal); Methodology (equal); Resources (equal); Software (equal); Writing‐review & editing (equal). **Yin Zheng:** Conceptualization (equal); Writing‐original draft (equal); Writing‐review & editing (equal). **Bin Chen:** Data curation (equal); Formal analysis (equal); Methodology (equal); Resources (equal); Writing‐review & editing (equal).

## Supporting information

Fig S1

Table S1‐S2

## Data Availability

Research data not shared.
