# Supplementary material for: The miRNA‐15b/USP7/KDM6B axis engages in the initiation of osteoporosis by modulating osteoblast differentiation and autophagy
Source: J Cell Mol Med. 2021 Jan 12;25(4):2069–81. doi: 10.1111/jcmm.16139 (PMC7882933; doi:10.1111/jcmm.16139)
Supplement: Supplementary file 2 — Table S1‐S2 [file JCMM-25-2069-s002.docx]

**Supplementary Table 1** Establishment of mouse model

| Order | Group | Teatment |
| --- | --- | --- |
| 1 | sham | sham-operated |
| 2 | Osteoporosis | ovariectomy |
| 3 | agomir-NC | ovariectomy, followed by injection of agomir-NC |
| 4 | miR-15b agomir | ovariectomy, followed by injection of miR-15b agomir |
| 5 | antagomir-NC | ovariectomy, followed by injection of antagomir-NC |
| 6 | miR-15b antagomir | ovariectomy, followed by injection of miR-15b antagomir |
| 7 | agomiR-NC+oe-NC | ovariectomy, followed by injection of agomir-NC+oe-NC |
| 8 | miR-15b agomir +oe-NC | ovariectomy, followed by injection of miR-15b agomir +oe-NC |
| 9 | miR-15b agomir + oe-KDM6B | ovariectomy, followed by injection of miR-15b agomir + oe-KDM6B |

**Supplementary Table 2** Osteoblast transfection

| Order | Group | Treatment | Effect |
| --- | --- | --- | --- |
| 1 | mimic-NC | Transfection of plasmids expressing mimic-NC | Positive control |
| 2 | miR-15b mimic | Transfection of plasmids expressing miR-15b mimic | Overexpress miR-15b |
| 3 | inhibitor-NC | Transfection of plasmids expressing inhibitor-NC | Negative control |
| 4 | miR-15b inhibitor | Transfection of plasmids expressing miR-15b inhibitor | Silence miR-15b |
| 5 | mimic-NC+Oe-NC | Transfection of plasmids expressing mimic-NC and oe-NC | Positive control |
| 6 | miR-15b mimic+Oe-NC | Transfection of plasmids expressing miR-15b mimic and oe-NC | Overexpress miR-15b |
| 7 | miR-15b mimic+Oe-USP7 | Transfection of plasmids expressing miR-15b mimic and oe-USP7 | Overexpress miR-15b and USP7 |
| 8 | oe-NC | Transfection of plasmids expressing oe-NC | Positive control |
| 9 | oe-USP7 | Transfection of plasmids expressing oe-USP7 | Overexpress USP7 |
| 10 | oe-NC+DMSO | Transfection of plasmids expressing oe-NC and treatment with DMSO | Negative control |
| 11 | oe-NC+MG132 | Transfection of plasmids expressing oe-NC and treatment with protease inhibitor MG132 | Negative control |
| 12 | oe-USP7+DMSO | Transfection of plasmids expressing oe-USP7and treatment with DMSO | Positive control |
| 13 | oe-USP7+MG132 | Transfection of plasmids expressing oe-USP7 and treatment with protease inhibitor MG132 | Experiment group |
| 14 | Oe-NC+si-NC | Transfection of plasmids expressing oe-NC and si-NC | Negative control |
| 15 | Oe-USP7+si-NC | Transfection of plasmids expressing oe-USP7 and si-NC | Positive control |
| 16 | Oe-USP7+si-KDM6B | Transfection of plasmids expressing oe-USP7 and si-KDM6B | Overexpress USP7 and silence KDM6B |
